# Insights Into the Characteristics of Sweet Syndrome in Patients With and Without Hematologic Malignancy

**DOI:** 10.3389/fmed.2020.00020

**Published:** 2020-02-18

**Authors:** Siting Zheng, Sheng Li, Shunli Tang, Yunlei Pan, Yuwei Ding, Jianjun Qiao, Hong Fang

**Affiliations:** Department of Dermatology, College of Medicine, The First Affiliated Hospital, Zhejiang University, Hangzhou, China

**Keywords:** Sweet syndrome, hematologic malignancy, treatment, outcome, mortality

## Abstract

**Background:** Sweet syndrome is a neutrophilic dermatosis that could be associated with malignancy, especially hematologic malignancy. Few studies have systematically elaborated on this disorder and its features related with hematologic malignancy.

**Objective:** This study aimed to describe the clinicopathological characteristics, treatment, and outcome of Sweet syndrome and to evaluate patient characteristics associated with hematologic malignancy.

**Methods:** We retrospectively reviewed patients with Sweet syndrome at the Department of Dermatology, the First Affiliated Hospital of Zhejiang University from October 2010 to February 2019.

**Results:** The study included 37 patients (16 men and 21 women), with a mean age of 53 years. Ten patients (27%) were classified as having malignancy-associated Sweet syndrome: nine with a hematologic malignancy including acute myeloid leukemia (4/9, 44%), myelodysplastic syndrome (4/9, 44%), and multiple myeloma (1/9, 11%) and one with a solid tumor diagnosed with liver carcinoma. The mean hemoglobin and platelet levels (*P* = 0.007 and *P* = 0.013, respectively), were significantly lower in patients with hematologic malignancy than in those with Sweet syndrome only. No significant difference in histopathology was found between patients with and without hematologic malignancy. Systemic corticosteroids were the most frequently used treatment (24/37, 65%). Higher mortality was found in patients with hematologic malignancy.

**Conclusion:** It is important to assess Sweet syndrome patients who have laboratory evidence of lower hemoglobin and platelet levels for a hematologic malignancy.

## Introduction

Sweet syndrome, also known as acute febrile neutrophilic dermatosis, is characterized by tender erythematous plaques, papules, or nodules most commonly involving the extremities, neck, and head. Histopathological findings include dense neutrophilic dermal infiltrate with edema (1). Sweet syndrome can be further classified into three subgroups as classic, malignancy associated, and drug induced (2). The diagnostic criteria for Sweet syndrome were proposed by Su and Liu and modified by von den Driesch in 1994 (3, 4). Acute myeloid leukemia (AML) was reported to be the most common hematologic malignancy associated with Sweet syndrome (5). Sweet syndrome in patients with AML may show atypical clinical forms with an aggressive course (6–8).

The objective of this study was to identify characteristics in Sweet syndrome associated with hematologic malignancy by comparing the clinicopathological features of Sweet syndrome with and without hematologic malignancy.

## Methods

### Patients and Data Collection

We retrospectively reviewed patients with Sweet syndrome at the First Affiliated Hospital of Zhejiang University between October 1, 2010 and February 28, 2019. The patients were diagnosed as having Sweet syndrome based on clinical, histopathological, and laboratory studies. After the approval of the ethics committee of the First Affiliated Hospital, College of Medicine, Zhejiang University (February 22, 2018; approval number 2018–122), their medical records were reviewed. From the medical records, we accessed demographic data, cutaneous lesion distribution and morphology, clinical symptoms, laboratory and histopathological findings, comorbid diseases, and treatment. All skin biopsy specimens were examined by two experienced dermatopathologists.

### Statistical Analysis

Continuous variables were summarized with means and standard deviations. Categorical variables were reported as proportions and percentages. Comparison between the groups was evaluated by using logistic regression. *P*-values of < 0.05 were considered statistically significant. All statistical analyses were performed in IBM SPSS 25.

## Results

### Demographic Data

Thirty-eight patients with Sweet syndrome were identified between October 1, 2010, and February 28, 2019, by reviewing the medical records in our center. One patient was excluded for lack of information. Therefore, the data of 37 patients were analyzed. Of the 37 patients, 21 (57%) were female and 16 (43%) were male. The mean age at presentation of Sweet syndrome was 53 years (range, 24–82 years) (Table 1). All the patients in our cohort were Chinese, and 26 (70%) presented in the inpatient setting. Ten patients satisfied the Curth postulates (9), of whom nine had an associated hematologic malignancy and one had a solid tumor. According to the results of bone marrow aspiration and biopsy, associated hematologic malignancies were identified as AML (4/9, 44%), myelodysplastic syndrome (MDS; 4/9, 44%), and multiple myeloma (MM; 1/9, 11%). One patient with a solid tumor was diagnosed with liver carcinoma by histopathology examination after the surgical resection.

**Table 1:** Sweet syndrome in patients with and without hematologic malignancy.

**Characteristic**	**Hematologic malignancy (*n* = 9)**	**Without hematologic malignancy (*n* = 28)**	***P*-value**
Age, years			
Mean (SD)	51 (13)	54 (12)	0.560
Median (range)	52 (24-71)	53 (28-82)	
Female, *n* (%)	5 (56)	16 (57)	0.933
Fever, *n* (%)	7 (78)	17 (61)	0.494
Cough/myalgia/fatigue, *n* (%)	2 (22)	6 (21)	0.958
Arthralgia, *n* (%)	0 (0)	2 (7)	0.999
Mucosal lesions, *n* (%)	3 (33)	6 (21)	0.473
Oral ulcers/blisters	3 (33)	4 (14%)	
Vulvar ulcers	0	1 (4)	
Conjunctivitis	0	1 (4)	
Generalized lesions, *n* (%)	6 (67)	18 (64)	1.000
Pain, *n* (%)	6 (67)	15 (54)	0.637
Pruritus, *n* (%)	1 (11)	8 (29)	0.267
**Laboratory findings**			
Hemoglobin [g/L, mean (SD)]	77.6 (14.0)	118.0 (24.8)	0.007
Platelet count [10^9^/L, mean (SD)]	94.1 (100.8)	280.4 (137.4)	0.013
Leukocyte count [10^9^/L, mean (SD)]	8.8 (7.7)	9.2 (4.5)	0.852
Erythrocyte sedimentation rate [mm/h, mean (SD)]	90.0 (45.7)	58.2 (34.6)	0.148

### Clinical and Laboratory Findings With and Without Hematologic Malignancy

Table 1 shows a comparison of Sweet syndrome between the patients with and without hematologic malignancy. Hematologic malignancy was associated with decreased levels of hemoglobin (*P* = 0.007) or platelet (*P* = 0.013) (Table 2). No statistically significant differences in age, sex, fever, cough, myalgia/fatigue, arthralgia, mucosal involvement, distribution of lesions, pain, pruritus, leukocyte count, and erythrocyte sedimentation rate (ESR) were found. In the hematologic malignancy group, mucosal lesions were oral ulcers/blisters, whereas in the other group, mucosal involvement included oral ulcers/blisters, vulvar ulcers, and conjunctivitis. Only two patients in the group without hematologic malignancy had arthralgia.

**Table 2:** Comparison of hemoglobin and platelet levels between Sweet syndrome patients with and without AML/MDS [mean (SD)].

	**AML (*n* = 4)**	**MDS (*n* = 4)**	**No AML/MDS (*n* = 29)**	*P*-value (AML^*^, MDS^*^)
Hemoglobin (g/L)	67.8 (5.7)	83.3 (14.9)	118.7 (25.7)	0.038, 0.046
Platelet count (10^9^/L)	123.8 (149.1)	53.5 (29.3)	269.7 (137.0)	0.093, 0.046

AML^*^, Comparison of hemoglobin and platelet levels between Sweet syndrome patients with AML and without AML/MDS; MDS^*^, Comparison of hemoglobin and platelet levels between Sweet syndrome patients with MDS and without AML/MDS.

### Histopathological Findings

In terms of histopathological features, the main finding was a predominantly neutrophilic dermal infiltrate (37/37, 100%) and prominent dermal papillary edema (26/37, 70%). The cell infiltrate was usually diffuse (30/37, 81%). All the cases had an infiltrate in the superficial dermis, 51% (19/37) had involvement in the mid-dermis, and 38% (14/37) had involvement in the deep dermis, even the subcutis. Lymphocytes (10/37, 27%), histiocytes (8/37, 22%), eosinophils (4/37, 11%), and plasmacytes (4/37, 11%) were the other cells involved in the infiltrate. Subepidermal vesicles (5/37, 14%) and ulcers (4/37, 11%) were also found. We observed malignant cells in the dermis in one patient with Sweet syndrome and AML.

### Treatment and Outcome

Of 37 patients, 24 (65%) received systemic corticosteroids. Antibiotics were the second common treatment used to treat underlying infections or as prophylaxis in 11 patients (30%). Other utilized drugs were thalidomide (1/37, 3%) and triptolide (1/37, 3%). In the hematologic group, patients were given systemic corticosteroids (7/9, 78%) or antibiotics (2/9, 22%), while in the other cases, patients were given systemic corticosteroids (16/28, 61%), antibiotics (9/28, 32%), thalidomide (1/28, 4%), and triptolide (1/28, 4%). All patients showed improvement after receiving therapy. Although lesions were localized or generalized, there seemed to be no significant difference in response.

In the hematologic malignancy group, three cases died from AML (3/9, 33%), and one case died from MDS (1/9, 11%), whereas in the group of patients with Sweet syndrome only, the mortality was 14%.

## Discussion

In this study, we observed that Sweet syndrome was more frequent in the women, and the mean age at diagnosis was 53 years, consistent with the data described in the literature (10–13). Our results failed to show significant differences in age and sex in the patients of Sweet syndrome with and without hematologic malignancy.

A retrospective case series of 83 Sweet syndrome patients with and without malignancy corroborated that leukopenia, anemia, and thrombocytopenia were associated with malignancy (14). Our study also revealed that hematologic malignancy was associated with lower hemoglobin and platelet levels. Concerning lower hemoglobin and platelet levels, which are usually the major phenomena found in MDS but not in AML, we separately examined the hematological findings between AML and MDS. As shown in the results presented in Table 2, lower hemoglobin levels were associated with AML and MDS while lower platelet levels were mainly related to MDS.

Notably, Sweet syndrome occurred after the development of hematologic malignancy in most of the patients with hematologic malignancy (7/9, 78%). Thus, we infer that lower hemoglobin and platelet levels might be a manifestation of AML/MDS or a therapeutic response for hematologic malignancy. ESR, which is known as an inflammatory marker, was higher in the patients with hematologic malignancy. Casarin Costa et al. first demonstrated the association between malignancy and higher ESR levels in patients with Sweet syndrome (13).

In our cohort, fever was notably more common than other clinical symptoms (24/37, 65%), with a higher incidence than that in some studies (11–13), emphasizing that fever exists as an important diagnostic criterion of Sweet syndrome (3, 4). Our study obtained a positive association with arthralgia in only 5% of the patients, none of whom had Sweet syndrome with hematologic malignancy. Although some researchers revealed that absence of arthralgia was associated with malignancy (14), our study did not reach a consistent conclusion.

The associated hematologic malignancies in this study included AML, MDS, and MM. The most common hematologic malignancies were AML and MDS (both in 11% of the patients), followed by MM (in 3%). The incidence of AML in patients with Sweet syndrome was 4% (10), which was lower than the incidence reported in our cohort. Sweet syndrome can present at various stages of malignancy, including before, after, or at the diagnosis of malignancy (7), which suggests that Sweet syndrome may appear as an indicator of underlying malignancy. Among the nine patients with hematologic malignancy, Sweet syndrome was identified after the diagnosis of hematologic malignancy in 78% of the patients, which demonstrates that Sweet syndrome can also occur as a paraneoplastic condition in patients previously diagnosed as having hematologic malignancy (15).

We detected the mortality as 44% in the group of cases associated with hematologic malignancy, compared with 14% in patients without hematologic malignancy. Reviewing the literature, few researchers compared the mortality or prognosis between these two groups mainly due to loss of follow-up. According to the follow-up documentation about death in this study, we noticed that in the hematologic malignancy group, three cases died from AML (3/9, 33%) and one case died from MDS (1/9, 11%). One previous study reported that the median overall survival in Sweet syndrome patients with AML was not significantly different from AML patients without Sweet syndrome (7). Therefore, we consider mortality/prognosis to be determined by the hematologic malignancy rather than the Sweet syndrome.

A recent retrospective study showed the histiocytoid or subcutaneous variants exclusively in the setting of malignancy (14). Several studies have also suggested the relationship of histiocytoid or subcutaneous Sweet syndrome with hematologic disorders (16–19). However, our cases did not show statistically significant differences in histopathology between the patients with and without hematologic malignancy. Alegria-Landa et al. observed that authentic histiocytes replaced previous neutrophilic infiltrates at the late-stage lesion of conventional Sweet syndrome (16). Therefore, we consider that the sample size, location, and time of skin biopsy may partially explain this difference from prior studies. Of the nine patients with hematologic malignancy, malignant cells (leukemic cells) in the dermal infiltrate were detected in only one patient, which can be defined as a coexistence of leukemia cutis and Sweet syndrome. Whether a skin infiltrate can be diagnosed as leukemia cutis may only be ascertained through clinical long-term follow-up.

A review about Sweet syndrome that was published in 2019 categorized corticosteroids and other agents such as potassium iodide or colchicine as the first-line treatments (20). Tumor necrosis factor α (TNF-α) antagonists and interleukin 1 (IL-1) inhibitors have also been reported to be effective (21–27). However, the data supporting the effectiveness of these biological agents are from case reports and small studies. In our case series, systemic corticosteroids and antibiotics were the most common medications for treatment. Antibiotic therapy may be used for treating underlying infections or as prophylaxis, especially during the chemotherapy period, rather than the Sweet syndrome.

As the retrospective study design is a limitation, the results of this study must be interpreted with caution. The small sample size, different follow-up times, and disease progression also affect the applicability of the results of the present study. Another limitation was the lack of analysis concerning cytogenetic or molecular aberrations in the patients with Sweet syndrome and hematologic malignancy.

## Conclusion

Lower hemoglobin and platelet levels were associated with hematologic malignancy, which suggests the importance of screening for potential hematologic malignancy when treating patients with Sweet syndrome. The mortality of patients with hematologic malignancy was higher than that of patients without hematologic malignancy as the cause of death is most probably hematologic malignancy.

## Data Availability Statement

The datasets generated for this study are available on request to the corresponding author.

## Author Contributions

SZ, JQ, and HF contributed to the conception and design of the study. SZ collected clinical data. SL and ST performed the statistical analysis. SZ wrote the first draft of the manuscript. YD and YP wrote sections of the manuscript. All authors contributed to manuscript revision and read and approved the submitted version.

### Conflict of Interest

The authors declare that the research was conducted in the absence of any commercial or financial relationships that could be construed as a potential conflict of interest.
